# Geochemistry, faunal composition and trophic structure in reducing sediments on the southwest South Georgia margin

**DOI:** 10.1098/rsos.160284

**Published:** 2016-09-28

**Authors:** James B. Bell, Alfred Aquilina, Clare Woulds, Adrian G. Glover, Crispin T. S. Little, William D. K. Reid, Laura E. Hepburn, Jason Newton, Rachel A. Mills

**Affiliations:** 1 School of Geography and Water@Leeds, University of Leeds, Leeds LS2 9JT, UK; 2 School of Earth and Environment, University of Leeds, Leeds LS2 9JT, UK; 3 Life Sciences, Natural History Museum, Cromwell Road, London SW7 5BD, UK; 4 Ocean and Earth Science, National Oceanography Centre Southampton, University of Southampton, Southampton SO14 3ZH, UK; 5 School of Biology, Newcastle University, Ridley Building, NE1 7RU, UK; 6 NERC Life Sciences Mass Spectrometry Facility, SUERC, East Kilbride G75 0QF, UK

**Keywords:** methane, Southern Ocean, ecology, assemblage composition, trophodynamics, authigenic carbonates

## Abstract

Despite a number of studies in areas of focused methane seepage, the extent of transitional sediments of more diffuse methane seepage, and their influence upon biological communities is poorly understood. We investigated an area of reducing sediments with elevated levels of methane on the South Georgia margin around 250 m depth and report data from a series of geochemical and biological analyses. Here, the geochemical signatures were consistent with weak methane seepage and the role of sub-surface methane consumption was clearly very important, preventing gas emissions into bottom waters. As a result, the contribution of methane-derived carbon to the microbial and metazoan food webs was very limited, although sulfur isotopic signatures indicated a wider range of dietary contributions than was apparent from carbon isotope ratios. Macrofaunal assemblages had high dominance and were indicative of reducing sediments, with many taxa common to other similar environments and no seep-endemic fauna, indicating transitional assemblages. Also similar to other cold seep areas, there were samples of authigenic carbonate, but rather than occurring as pavements or sedimentary concretions, these carbonates were restricted to patches on the shells of *Axinulus antarcticus* (Bivalvia, Thyasiridae), which is suggestive of microbe–metazoan interactions.

## 1. Introduction

Cold seeps are discontinuous areas of methane and hydrogen sulfide seepage and are widespread in both shallow and deep water [1,2]. There has been much interest in submarine methane deposits, both for their potential as an energy source but also the threat posed to the climate by the potential release of this potent greenhouse gas [1,3]. Thus, the fate of methane in marine sediments is an area of interest for wider climate science [4–6]. Areas of methane seepage are commonly identifiable by high levels of dissolved methane, bubble plumes/gas flares or bottom simulating reflectors [7–10]. High latitude continental shelf regions are areas with potentially widespread methane storage, particularly in the Arctic and Southern Oceans, where the presence of methane reservoirs has been confirmed or inferred at a number of sites [7,9–17]. Since methane can provide a basis for chemosynthesis, this has clear implications for biogeochemical cycles and the range of available habitats and biodiversity in the polar oceans [18]. Methane-enriched, reducing sediments are part of a continuum between cold seeps and background sediments in the deep sea [19] and may support transitional faunal assemblages. Research into faunal composition and trophic structure in these settings is very limited and here we present for the first time a comprehensive study of geochemical and faunal characteristics of reducing sediments from the Southern Ocean.

Sediments that host an active methane flux to bottom water are often places of elevated biomass and abundance of deep-sea benthos [18,20,21], representing important, localized sources of organic matter for benthic food webs. Such sediments are characterized by elevated methane and hydrogen sulfide concentrations. Sulfide is produced through sulfate reduction during anaerobic oxidation of methane (AOM) [22–25] and can in turn supply reduced chemical species for chemosynthetic reaction pathways that depend upon sulfide oxidation [18,26–28]. Methane-derived carbon (MDC) is an important metabolic resource at high activity seeps [18,21,26,29,30], but the influence of methane-enriched sediments upon trophic structure in areas of lower methane flux is comparatively unknown. MDC is commonly associated with very low δ^13^C signatures (typically −100 to −40‰) compared with other sources of organic matter (typically more than −30‰) [20,30]. AOM can also yield very low δ^34^S signatures as a result of sulfate reduction making stable isotope analysis (SIA) an ideal tool to estimate the extent of MDC utilization [24]. Carbon (δ^13^C : δ^12^C) and sulfur (δ^34^S : δ^32^S) isotope ratios do not fractionate much between trophic levels and so are useful in detecting differences in food source partitioning. Nitrogen (δ^15^N : δ^14^N) isotope ratios provide information on trophic level of individuals or species within a food web as its isotopic signature is increased (approx. 3‰ but can vary widely) during the transfer of proteins from source to consumer [30–32]. Phospholipid fatty acids (PLFAs) can also provide useful insights into the relative abundance and isotopic signatures of certain bacterial biomarkers [33–35]. These compounds degrade quickly following death and so can provide a useful insight into recent microbial activity.

Highly active seeps, typically identified as areas with gas flares or dense aggregations of seep-endemic fauna, have been studied in a number of areas [18,20,21,36,37]. However, the extent, ecology and biogeochemistry of sediments with elevated levels of methane but not sufficiently deep [38] or methane-enriched to support seep-endemic fauna, is not clear. It is possible that, through ocean warming, submarine methane hydrate deposits, which are globally widespread [17,39] will gradually destabilize as bottom temperatures increase, further increasing the extent of such environments [40]. This investigation of the geochemical and biological processes in methane-enriched sediments provides a basis for estimating possible future ecological scenarios.

## 2. Aims and hypotheses

**Figure 1.**
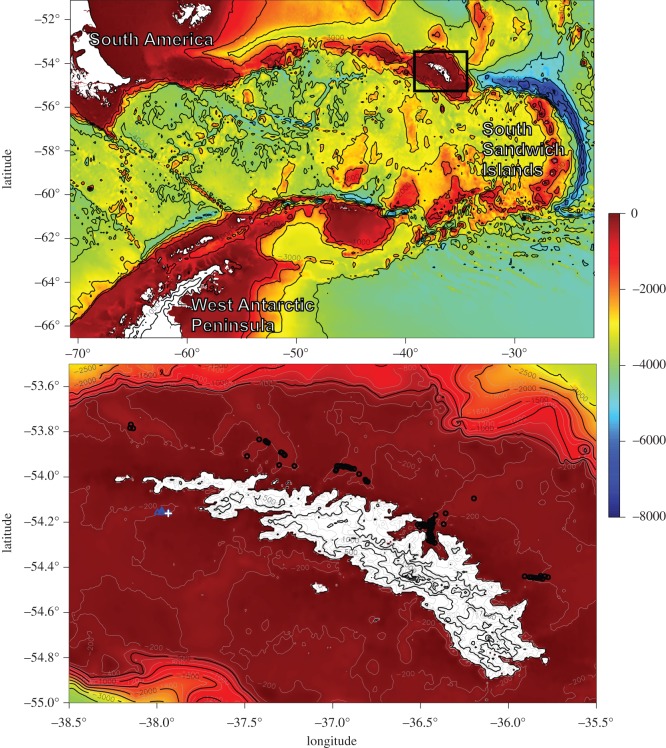
Map of sampling locations around South Georgia. Blue triangles indicate JC42 and JC55 sampling stations. Black circles indicate positions of gas flares [10] and the white cross shows the location of recovered ikaite crystals [41]. Bathymetry data from GEBCO.

Around the sub-Antarctic island of South Georgia (54° S), numerous cold seep sites have been identified in fjords along the northern margin [10] (figure 1). We present a combined geochemical and faunal study of reducing sediments on the southwest South Georgia margin, surveyed during two separate expeditions in 2010 and 2011 [15,16] in order to investigate the role of reducing conditions in marine sediment and its impact upon faunal communities with a view to addressing the following hypotheses: (1) faunal community composition is controlled by the extent of reducing conditions, (2) MDC is a significant basal component of the food web and (3) sediments on the South Georgia margin show geochemical signatures consistent with methane seepage.

## 3. Material and methods

### 3.1. Sampling

**Figure 2.**
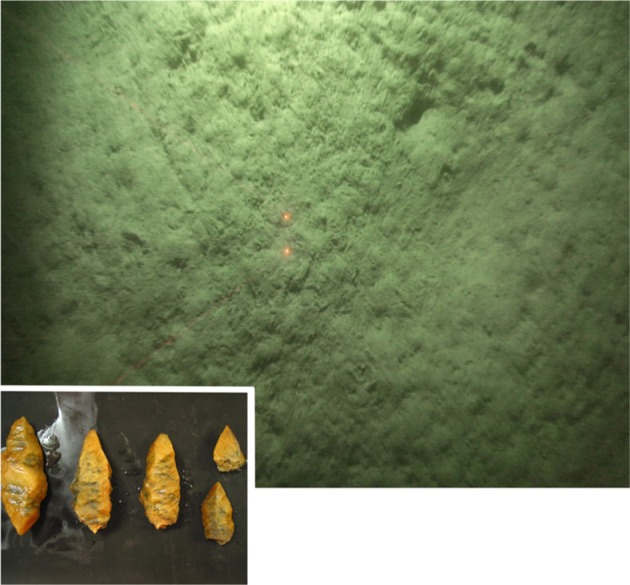
Images of seafloor of survey region from towed video platform and crystals of Ikaite recovered [41]. Crystal image courtesy of Mark Belchier. Red laser dots 10 cm apart.

An area of potential methane seepage was originally identified on the southwest South Georgia margin [41]. During a routine fisheries survey, friable samples of what were initially identified as methane hydrate were recovered from a trawl of sulfidic sediments at around 250 m depth (figure 1; [41]). Upon closer inspection, and cross-referencing with environmental conditions, it became apparent that these samples were not methane hydrate but were in fact the metastable authigenic carbonate ikaite (Belchier & Larter 2016, personal communications (figure 2)). The presence of authigenic carbonates, such as ikaite, is sometimes associated with methane seepage [2,42,43], and the area was identified as a site of interest for the study of Southern Ocean marine chemosynthetic ecosystems, though unfortunately the ikaite crystals quickly degraded after recovery and could not be preserved. During the ‘Chemosynthetic Ecosystems South of the Polar Front’ research programme, the area was visited during two separate expeditions: RRS *James Cook* 42 (JC42, Jan–Feb 2010) and 55 (JC55, Jan–Feb 2011). We report data from sediment geochemistry samples, taken during JC42 and JC55 [15,16], and faunal samples taken during JC55 [16]. We visited three stations on the southwest South Georgia margin (table 1; figure 1) at around 250 m depth, distributed over an area of approximately 7 km^2^.

**Table 1.** Positions (degrees and decimal minutes), depths and usage of JC42 gravity core and JC55 megacore deployments on the southwest South Georgia margin. Three to four cores pooled per deployment for quantitative macrofauna. XRD, X-ray diffraction; SIA, stable isotope analysis; PLFA, phospholipid fatty acids.

cruise	station	sub-station	latitude	longitude	depth (m)	faunal preservation	use(s)
JC42	02_03	1	−54.1575	−37.9761	257	n.a.	Geochemistry
JC55	111	1	−54.1575	−37.9761	257	n.a.	Dissolved O_2_, PLFAs
	112	1	−54.1575	−37.9761	257	6% Form.	Quant. Macrofauna, Geochemistry, XRD
	113	2	−54.1580	−37.9345	247	80% Etoh	Macrofaunal SIA, Geochemistry, XRD
	117	3	−54.1475	−37.9717	254	6% Form.	Quant. Macrofauna, Geochemistry, Dissolved O_2_
	118	2	−54.1580	−37.9344	247	6% Form.	Quant. Macrofauna, XRD
	119	2	−54.1580	−37.9344	248	80% Etoh	Macrofaunal SIA
	120	2	−54.1580	−37.9344	248	6% Form.	Quant. Macrofauna, XRD
	121	2	−54.1580	−37.9344	248	6% Form.	Quant. Macrofauna
	122	2	−54.1580	−37.9344	248	80% Etoh	Macrofaunal SIA, XRD

### 3.2. Fauna

Fauna were all sampled during JC55 using a Bowers–Connelly dampened megacorer fitted with eight 10 cm diameter tubes [44]. Between one and six deployments were taken from each site (table 1) giving a total area quantitatively sampled of approximately 1490 cm^2^. Between three and four individual cores were pooled from each corer deployment, as cores from the same deployment are pseudoreplicates [45]. Material retained on a 300 µm sieve was either preserved directly in 10% formalin (in buffered seawater) for quantitative community analyses or live sorted and subsequently preserved in 80% ethanol for on-board photographic documentation and SIA. All faunal samples were sorted under a dissecting microscope to either species/morphospecies level (annelid and bivalve taxa) or higher levels for low abundance taxa (e.g. family level for peracarid crustaceans). Species identities were cross-referenced with the Ocean Biogeographic Information System (OBIS).

### 3.3. Stable isotopes

A combination of dual- (δ^13^C and δ^15^N, 91 samples) and tri- (δ^13^C, δ^15^N and δ^34^S, 35 samples) isotope techniques was used to describe isotopic signatures of 29 species/morphospecies of macrofauna, sediment organic matter content and profiles of sediment organic carbon. The number of faunal samples submitted for C/N and C/N/S analyses were representative of 88 and 92% of the total faunal abundance, respectively, including the 12 most abundant taxa (1–10 replicates per taxa depending upon abundance/availability of sample mass). Specimens submitted for isotope analyses were pooled if necessary to achieve a specified optimal mass (C/N = 0.7 mg ± 0.5, C/N/S = 2.5 mg ± 0.5). Where possible, individual specimens were submitted as separate samples in order to preserve variance structure within populations [46,47] but in some cases, low sample mass meant individuals had to be pooled (e.g. oligochaetes). Specimens were dried for a minimum of 24 hours at 50°C and weighed in mg (correct to 3 d.p.) into ultra-clean tin capsules and stored in a desiccator whilst awaiting SIA.

Subsamples of sediment were taken from two cores (112 and 117; table 1) for organic carbon profiles and freeze-dried. Sediment samples (approx. 1 mg) were acidified using 6 M HCl to remove inorganic carbon. Thus, δ^13^C analyses were acquired from sediment organic carbon (with the carbon content used to calculate percentage organic carbon). Surface samples of freeze-dried sediment from each site (0–1 cm below seafloor (cmbsf)) were also analysed for C/N/S isotopes (untreated for N/S and acidified with 6 M HCl for C) [40].

Only specimens preserved in ethanol were used for SIA in an attempt to regulate the influence of preservation effects. As a result, only faunal specimens from the southeast of the study area could be analysed for isotopic signatures (table 1). Specimens had any attached sediment removed prior to SIA and specimens with carbonate structures (i.e. bivalves) were physically removed from their shells. However, given the low sample mass available and the deleterious effects of acid upon nitrogen and sulfur ratios [48], these could not feasibly be acidified. The vast majority of the species that occurred in sufficient abundance for SIA were annelid taxa with no carbonate structures. A pilot study confirmed that acidification had little impact upon δ^13^C measurements, and it was considered more appropriate to preserve the integrity of nitrogen and sulfur isotope signatures [48,49] at the expense of a small increase in error for carbon isotope measurements. There are usually substantial differences in signatures of methanotrophic and photosynthetic C-fixation pathways, making mixed diets clearly detectable even with an additional δ^13^C error. δ^34^S measurements were also available to aid interpretation of diet.

All bulk elemental analyses were completed at the East Kilbride Node of the Natural Environment Research Council Life Sciences Mass Spectrometry Facility in 2015. Samples were analysed by continuous flow isotope ratio mass spectrometer using a Vario-Pyro Cube elemental analyser (Elementar), coupled with a Delta Plus XP isotope ratio mass spectrometer (Thermo Electron). Each of the runs of C/N and C/N/S isotope analyses used laboratory standards (gelatine and two amino acid–gelatine mixtures) as well as the international standard USGS40 (glutamic acid). C/N/S measurements used the internal standards (MSAG2: (methanesulfonamide/gelatine and methionine) and the international silver sulfide standards IAEA-S1, S2 and S3. All isotope measurements included samples of freeze-dried, powdered *Antimora rostrata* (ANR), an external reference used in other studies of chemosynthetic ecosystems [49,50]. This standard was used to monitor instrumental variation between runs, instruments and studies. Machine error for ANR measurements was 0.09, 0.35 and 0.93 for δ^13^C, δ^15^N and δ^34^S, respectively. Measurements of ANR were similar to previous studies, excepting δ^15^N from the tri-isotope run, and so these data were discarded. Stable isotope ratios are all reported in delta (δ) per mille (‰) notation, relative to international standards: V-PDB (δ^13^C); Air (δ^15^N) and V-CDT (δ^34^S).

### 3.4. Phospholipid fatty acids

Phospholipid fatty acid (PLFA) analyses were completed at the James Hutton Institute, University of Aberdeen using 3.38 g of freeze-dried surface (0–1 cm) sediment from megacore deployment 111 (table 1) following the procedure detailed in Main *et al.* [51], which we summarize below. Lipids were extracted following a method adapted from [52], using a single-phase mixture of chloroform : methanol : citrate buffer (1 : 2 : 0.8 v-v:v). Lipids were fractionated using 6 ml ISOLUTE SI SPE columns, preconditioned with 5 ml chloroform. Freeze-dried extract was taken up in 400 µl of chloroform, vortex mixed twice and allowed to pass through the column. Columns were washed in chloroform and acetone (eluates discarded) and finally 10 ml of methanol. Methanol eluates were collected in vials, allowed to evaporate under a N_2_ atmosphere and frozen at –20°C.

PLFAs were derivitized with methanol to produce fatty acid methyl esters (FAMEs). Samples were taken up in 1 ml of 1 : 1 (v : v) mixture of methanol and toluene. Then 1 ml of 0.2 M KOH (in methanol) was added with a known quantity of the C19 internal standard (nonadecanoic acid), vortex mixed and incubated at 37°C for 15 min. After cooling to room temperature, 2 ml of isohexane : chloroform (4 : 1 v:v), 0.3 ml of 1 M acetic acid and 2 ml of deionized water were added to each vial. The solution was mixed and centrifuged and the organic phase transferred to a new vial and the remaining aqueous phase was mixed and centrifuged again to further extract the organic phase, which was combined with the previous. The organic phases were evaporated under a N_2_ atmosphere and frozen at −20°C.

Samples were taken up in isohexane to perform gas chromatography–combustion–isotope ratio mass spectrometry (GC-C-IRMS). The quantity and δ^13^C values of individual FAMEs were determined using a GC Trace Ultra with combustion column attached via a GC Combustion III to a Delta V Advantage isotope ratio mass spectrometer (Thermo Finnigan, Bremen). The δ^13^C values (‰) of each FAME were calculated with respect to a CO_2_ monitoring gas, traceable to IAEA reference material NBS 19 TS-Limestone. Measurement of the Indiana University reference material hexadecanoicacid methyl ester (certified δ^13^C_VPDB_ 30.74 ± 0.01‰) gave a value of 30.91 ± 0.31‰ (mean ± s.d., *n* = 51).

Combined areas of all mass peaks (*m/z* 44, 45 and 46), following background correction, were collected for each FAME. These areas, relative to the internal C19 standard, were used to quantify abundance of each FAME and related to the PLFAs from which they are derived [53]. Compounds were compared with previous studies to identify biomarkers of specific bacterial processes or groups [34,35,54–61]. Bacterial biomass was calculated using transfer functions from the total mass of four PLFAs (i14 : 0, i15 : 0, a15 : 0 and i16 : 0), estimated at 14% of total PLFA biomass. PLFA biomass is estimated at 5.6% of total bacterial biomass [33].

### 3.5. Geochemistry sampling

A gravity core (*ca* 200 cm long) was retrieved during JC42 from a water depth of 257 m (JC42 02_03; table 1). This was divided into 50 cm long sections using a circular saw on board, and a nylon wire was used to split each section in two vertically: one half was used for analysis and the other was archived at −20°C. During JC55, sediment samples were collected using a Bowers & Connelly Megacorer [44] equipped with multiple polycarbonate tubes (10 cm diameter). These sediment cores (18–27 cm in length) were retrieved from three sites at water depths of 247–257 m (table 1). Gravity core sections and sampled megacores were immediately transferred into a glove bag under oxygen-free (N_2_ atmosphere) and temperature-controlled conditions (*ca* 4–6°C). Gravity core sections were subsampled with a clean spatula at intervals of 5–10 cm. Sampled megacores were manually extruded (at intervals of 1–2 cm) into a polycarbonate ring and sectioned using a PTFE sheet that was cleaned with de-ionized water between samples. Bottom water temperatures were 1.2°C.

### 3.6. Geochemistry analysis

Dissolved methane in sediment samples was measured as soon as possible after core collection using the headspace vial method [62]. In brief, open-ended, graduated plastic syringes were used to measure an aliquot (3 ml) of wet sediment, which was then extruded into a 20 ml glass vial, followed by 5.0 ml of 1.0 M NaOH to terminate microbial activity. The vial was crimped shut, shaken vigorously for several minutes and left to stand for more than 1 hour. The headspace was then sampled by syringe and the methane was analysed by GC-FID (Agilent 7890A).

Sediment porosity was calculated from the loss of water after drying the sediment at 60°C assuming densities of 2.6 and 1.025 g cm^−3^ for the sediment and pore fluid, respectively [63].

Shipboard determinations of dissolved oxygen content in the upper sediment were carried out on a dedicated core from two sites (table 1). A Unisense OX50 micro-sensor was calibrated between O_2_-saturated and anoxic seawater solutions with temperature and salinity equivalent to bottom waters. Unisense micro-profiling apparatus and SensorTrace PRO software resolved oxygen content at 100 and 200 µm intervals downcore. Oxygen profiles were later surface-normalized to bottom water values determined from water samples taken from deployments near the seafloor to convert to μmol kg^−1^.

Pore water was separated from the sediment matrix by centrifugation at 12 000*g* at 4°C for 10 min under N_2_; the supernatant fluids were filtered under N_2_ through disposable 0.2 µm cellulose nitrate membrane syringe filters (Whatman, UK). Filtered pore waters were divided for H_2_S, total alkalinity (TA), dissolved sulfate and dissolved metals (including Fe and Mn). H_2_S, TA and dissolved sulfate were determined onboard and samples for dissolved metal analysis were acidified (pH < 2) by adding 2 µl of concentrated HCl (UpA, Romil) per 1 ml of sample, pending analysis at the National Oceanography Centre, Southampton. Elemental abundances in pore waters were determined by inductively coupled plasma (ICP)-atomic absorption spectroscopy (Perkin Elmer Optima 4300DV). Instrument precision was better than 2% and measurements of an artificial seawater standard (CRM-SW) were within 1% of the recommended values.

TA and H_2_S were measured immediately following pore water extraction: TA by titrating against 0.10 M HCl while bubbling nitrogen through the sample [64] and H_2_S using standard photometric procedures based on formation of methylene blue [65]. Sulfate was measured by ion chromatography (Dionex ICS2500), with reproducibility better than 2% (determined by repeat analysis of a seawater standard).

### 3.7*.* X-ray diffractometer analyses

During sorting, it was noted that the valves of *Axinulu*s *antarcticus* (Bivalvia: Thyasiridae; Zelaya, 2010) were often partially coated in deposits of yellow, superficially amorphous material, typically close to the antero-dorsal margin. To determine the composition of these deposits, samples were scraped off the shells of the specimens and analysed using an X-ray diffractometer (XRD). Two samples of these deposits were submitted, as well as one of freeze-dried sediment as a control (table 1). Owing to an instrument failure, the first shell deposit sample was analysed using a different instrument to the second shell deposit sample and sediment sample. The first shell deposit sample was analysed using a Bruker D8 diffractometer and a Lynxeye detector. The remaining samples were analysed using a Philips PW1050 diffractometer and a point detector. Both systems used a *θ*/−2*θ* goniometer and Cu K α-1 radiation.

### 3.8. Statistical analyses

The following analyses were conducted in the R environment [66], using the Vegan library (v. 2.0–8) [67] unless otherwise specified. Only quantitatively preserved cores were used in assemblage composition analyses. Abundance data from quantitative cores was standardized to individuals per square metre. Community composition from these sites were compared to the composition of Antarctic shelf sediments and cold seep sites in the Pacific [68–70] using the metaMDS and PERMANOVA routine (999 permutations) [71]. Estimated species richness (100 individuals, 999 permutations) was calculated in EstimateS (v. 9.1.0) [72].

## 4. Results

### 4.1. Faunal community composition

A total of 4413 animal specimens were counted. Polychaetes dominated all samples (49–70%) with oligochaetes (15–34%) and bivalves (10–12%) accounting for most of the remaining taxa. The most abundant species was *Aphelochaeta glandaria* (Polychaeta: Cirratulidae; Blake, 1996) (1556 ind. counted, up to 14 420 ind. m^−2^), but also occurring in significant abundances were *Tubificoides* sp. (Oligochaeta: Tubificidae; Lastočkin, 1937) (500 ind. counted, up to 8531 ind. m^−2^), *Psamathe fauveli* (Polychaeta: Hesionidae; Averincev, 1962) (664 ind. counted, up to 5178 ind. m^−2^) and *Axinulus antarcticus* (Bivalvia) (456 ind. counted, up to 3756 ind. m^−2^) (table 2). Many specimens of *A. antarcticus* had an unidentified hydrozoan attached to the dorsal margin. These hydrozoan specimens fragmented and detached from their hosts and as such their abundance could not be quantified.

**Table 2.** Macrofaunal assemblage composition (quantitative cores only, abundance per m^2^) and stable isotopic data. A = 0–5 cmbsf, B = 5–10 cmbsf.

taxa			core				
class	genus/other	species	112	117	118	120	121
Polychaeta	*Parougia*	spA	95.50	466.87	318.32	31.83	445.64
	*Scoletoma*	*tetraura*	286.49	721.52	190.99	413.81	477.48
	Aphroditidae	Aphroditidae sp.	95.50	0.00	0.00	0.00	0.00
	Polynoidae	Polynoidae sp.	0.00	0.00	0.00	31.83	0.00
	Sigalionidae	Sigalionidae sp.	0.00	0.00	31.83	0.00	0.00
	Chrysopetalidae	Chrysopetalidae sp.	0.00	127.33	127.33	31.83	31.83
	*Psamathe*	*fauveli*	1305.10	5177.96	3215.01	2864.86	4169.96
	*Exogone*	(*parexogone*) *tasmanica*	509.31	594.19	477.48	286.49	445.64
	*Sphaerosyllis*	*hirsuta*	572.97	1485.48	732.13	700.30	954.95
	Syllides	sp.	286.49	212.21	127.33	63.66	95.50
	*Aglaophamus*	*trissophyllus*	0.00	84.88	0.00	0.00	0.00
	*Sphaerodoropsis*	*parva*	541.14	933.73	381.98	254.65	509.31
	*Sphaerodoropsis*	*arctowskyensis*	63.66	0.00	0.00	0.00	0.00
	Sphaerodorum	spA	31.83	42.44	0.00	0.00	63.66
	Phyllodocidae	Phyllodocidae sp.	0.00	42.44	0.00	0.00	0.00
	Apistobranchidae	Apistobranchidae sp.	31.83	42.44	63.66	31.83	31.83
	*Laonice*	*wedellia*	0.00	0.00	0.00	63.66	31.83
	*Spiophanes*	*kroeyeri*	222.82	42.44	350.15	159.16	127.33
	Spionidae	Spionidae sp.	0.00	0.00	31.83	0.00	0.00
	*Aphelochaeta*	*glandaria*	5634.22	10 483.26	14 419.78	10 122.50	11 491.26
	*Aphelochaeta*	*monilaris*	1209.61	169.77	318.32	1145.94	604.80
	*Tharyx*	*marioni*	0.00	169.77	0.00	63.66	254.65
	*Aphelochaeta*	sp. indet/juveniles	0.00	0.00	445.64	572.97	222.82
	*Diplocirrus*	*becci* sp. nov	413.81	551.75	63.66	31.83	159.16
	Sternaspidae	Sternaspidae sp.	0.00	169.77	0.00	0.00	31.83
	Ampharetidae	sp. indet	31.83	0.00	0.00	0.00	0.00
	*Glyphantostomum*	sp.	31.83	42.44	0.00	0.00	0.00
	Polycirrus	sp. indet/juveniles	31.83	0.00	31.83	0.00	0.00
	*Hauchiella*	*tribullata*	63.66	42.44	159.16	63.66	254.65
	Polycirrus	sp.	0.00	42.44	0.00	0.00	0.00
	Terebellidae	sp.	31.83	0.00	0.00	0.00	0.00
	Trichobranchidae	Trichobranchidae sp.	222.82	84.88	0.00	31.83	0.00
	*Capitella*	spA	0.00	0.00	0.00	159.16	0.00
	*Capitella*	spB	0.00	0.00	0.00	0.00	31.83
	*Maldane*	*sarsi antartica*	31.83	0.00	63.66	0.00	63.66
	*Clymenella*	*antarctica*	31.83	42.44	63.66	0.00	0.00
	Opheliidae	Opheliidae sp.	0.00	0.00	95.50	31.83	31.83
	*Leitoscoloplos*	*kerguelensis*	0.00	84.88	31.83	0.00	0.00
	*Leitoscoloplos*	*kerguelensis minutus*	31.83	0.00	0.00	0.00	0.00
	*Aricidea*	*antarctica*	668.47	679.08	859.46	350.15	1018.62
	*Levinsenia*	*gracilis*	31.83	84.88	159.16	0.00	95.50
	*Scalibregma*	*inflatum*	0.00	0.00	0.00	95.50	95.50
Bivalvia	*Axinulus*	*antarcticus*	2864.86	3225.62	3215.01	2260.05	3756.15
	*Propeleda*	sp. cf *longicaudauta*	63.66	169.77	0.00	95.50	95.50
	*Ennucula*	sp.	127.33	127.33	63.66	95.50	445.64
	*Mysella*	*antarctica*	63.66	0.00	31.83	0.00	63.66
Clitellata	*Limnodriloides*	spA	127.33	6069.25	1464.26	2801.19	2482.88
	*Tubificoides*	spA	8530.91	1612.81	2291.89	2164.56	1718.91
	*Limnodriloides*	spB	31.83	0.00	31.83	0.00	0.00
Malacostraca	Lysianassidae		0.00	42.44	31.83	0.00	0.00
	Phoxocephalidae		95.50	169.77	413.81	190.99	190.99
	Synopiidae		0.00	127.33	190.99	95.50	254.65
	Ampeliscidae		0.00	0.00	31.83	0.00	0.00
	Sphaeromatidae		0.00	0.00	31.83	0.00	0.00
	Idoteidae		0.00	0.00	63.66	0.00	31.83
	Antarcturidae		0.00	42.44	0.00	31.83	0.00
	Gynodiastylidae		0.00	42.44	63.66	0.00	0.00
	Pseudocumatidae		0.00	42.44	31.83	0.00	63.66
	Lampropridae		0.00	0.00	31.83	0.00	0.00
	Mysida		0.00	84.88	31.83	63.66	31.83
	Tanaidae		0.00	0.00	0.00	0.00	63.66
	Neotanaidae		95.50	127.33	63.66	0.00	0.00
Ophiuroidea	Ophiurina		31.83	42.44	31.83	0.00	0.00
Holothiuroidea	Holothuroidea		0.00	0.00	0.00	0.00	31.83
Ascidiacea	Ascidiacea		127.33	84.88	190.99	0.00	0.00
Annelida	Echiura	spB	31.83	0.00	0.00	0.00	31.83
	Sipuncula		700.30	509.31	445.64	190.99	604.80

Alpha diversity and estimated species richness were qualitatively similar in all deployments (table 3). Sub-station 1 had the greatest proportion of oligochaetes (34%, compared with 15–22% elsewhere) and the lowest proportion of polychaetes (49%, compared with 64–70% elsewhere). No significant differences were observed in the composition between each of the sub-stations (PERMANOVA, *F* = 0.79, *p* = 0.795).

**Table 3.** Faunal abundance, diversity and species richness of macrofauna from quantitatively sampled cores. Proportion of individuals counted to total density varied as a result of different numbers of pooled cores between deployments.

JC55 station	sub-station	Ind. counted	Ind. m^−2^	species observed	diversity (H’)	estimated species richness (*n* = 100) (±s.d.)
112	1	798	25 402	40	2.27	16.81 (2.55)
117	3	828	35 142	43	2.38	17.60 (2.56)
118	2	990	31 513	45	2.18	17.57 (2.43)
120	2	804	25 593	34	2.18	15.85 (2.24)
121	2	993	31 609	41	2.36	18.23 (2.36)

### 4.2. Isotopic analyses

**Figure 3.**
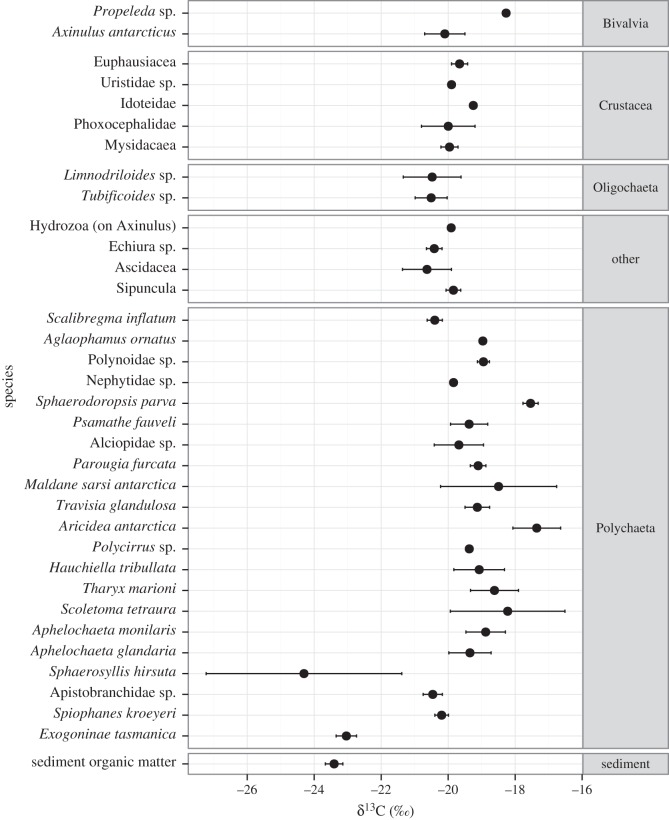

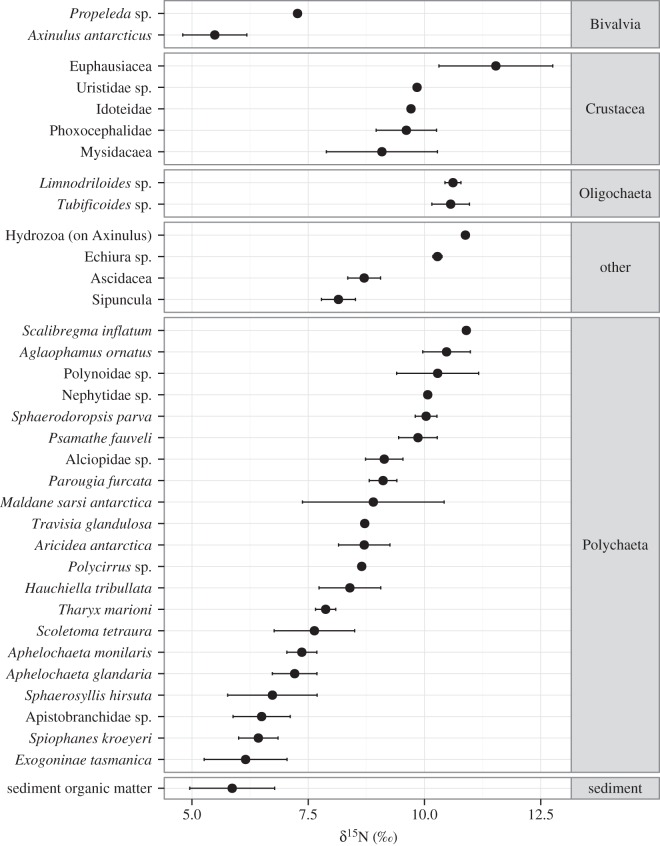
Species and sediment organic matter δ^13^C and δ^15^N ± 1 s.d., grouped by higher taxa.

**Figure 4.**
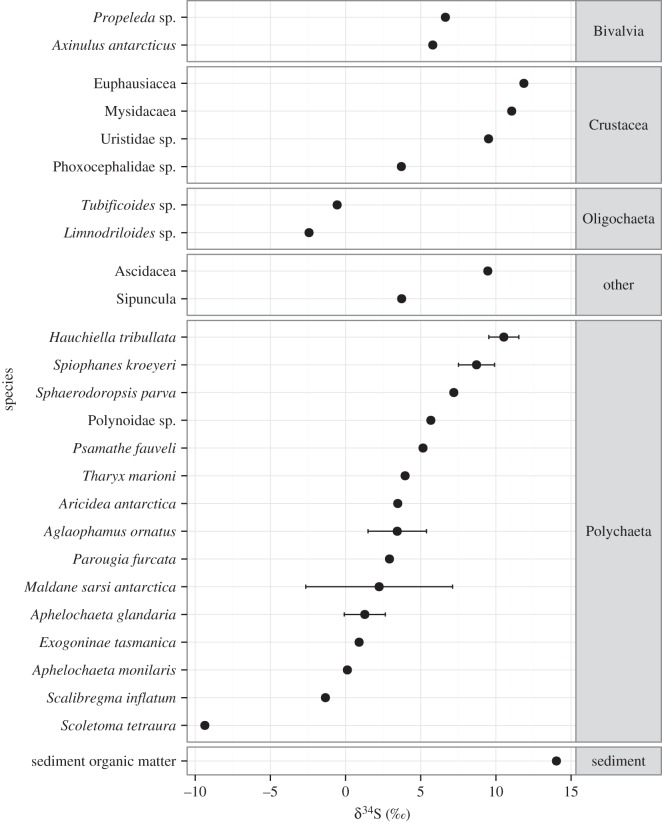
Faunal and Sediment organic matter δ^34^S ± 1 s.d., grouped by higher taxa.

Since only ethanol-preserved specimens were used for isotopic measurements, all faunal samples were from the second sub-station (table 1). Measurements of δ^13^C ranged from −26.37 to −15.87‰ across all faunal samples (mean: −19.55‰ ± s.d. 1.48). δ^15^N values ranged from 4.20 to 12.40‰ (mean: 8.28‰ ± s.d. 1.68; figure 3). Sediment organic material had low δ^15^N values (5.87‰ ± 0.91), indicating that it was the base of the food chain for most species. Several species also had a low δ^15^N signature similar to sediment organic matter (figure 3), suggesting an additional ^15^N-depleted source of organic nitrogen. All faunal δ^34^S signatures were less than that of surface sediment organic matter (OM) (14.03 ± 0.12‰) and varied widely (δ^34^S mean: 4.25‰ ± s.d. 5.13, −9.36–14.11‰), indicating at least two sources of organic sulfur (figure 4).

### 4.3. Poly-unsaturated fatty acids

A total of 34 compounds were identified, ranging in concentration between 0.006 and 1.637 µg per g freeze-dried sediment. These comprise 15 saturated fatty acids, 14 mono-unsaturated fatty acids (MUFAs) and 5 poly-unsaturated fatty acids. Most of the compounds occurred in low abundance (16 < 1% of total, 29 < 5%). The most abundant PLFAs were: C16:0 (1.637 µg g^–1^ sediment, 16.56% of total); C16:1ω7c (1.509 µg g^–1^, 15.39%); C18 : 1ω(10 or 11) (1.301 µg g^–1^, 13.51%) and C18 : 1ω7 (1.188 µg g^–1^, 12.34%). Carbon isotopic signatures of PLFAs ranged between −45.60 and −23.85‰ (δ^13^C mean −31.17‰). Bacterial total biomass was estimated from the concentration of i14 : 0, i15 : 0, ai15 : 0 and i16 : 0 at 113.14 µg g^–1^ sediment (115.97 µg cm^–3^).

### 4.4. Geochemistry

**Figure 5.**
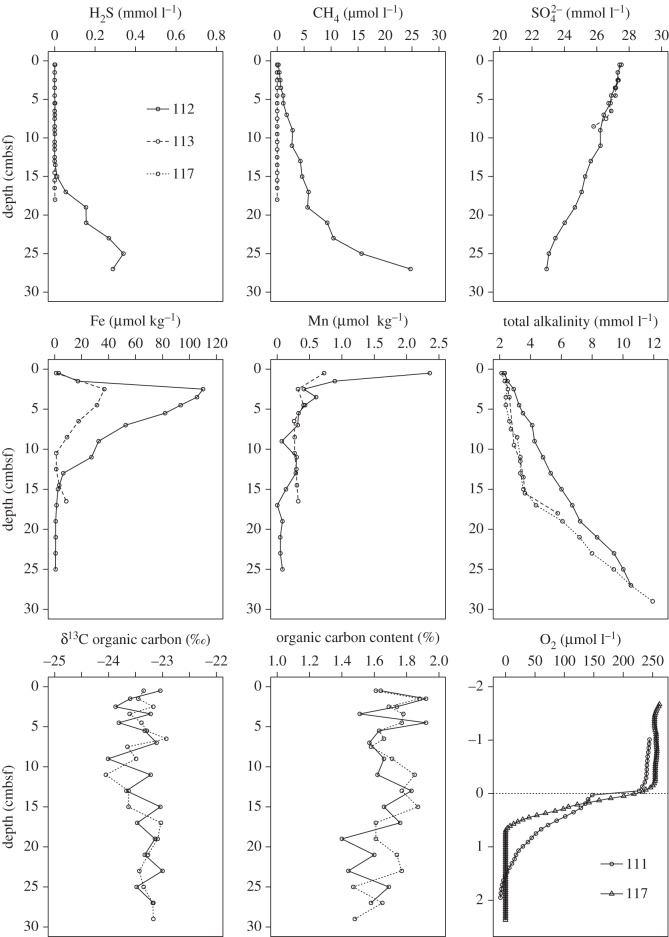
JC55 Megacore profiles. Legend as upper-left for all plots except oxygen (bottom-right).

Overall, downcore profiles are characterized by distinct geochemical zones indicative of early diagenesis (figure 5). A rapid downcore decrease in dissolved oxygen concentrations occurred in the top 0–2 cm, consistent with aerobic oxidation of organic material. Beneath the oxic layer (more than 1–2 cmbsf; figure 5), in the manganiferous and ferruginous zones, concentrations of dissolved Mn and Fe were elevated, reaching maximum concentrations of 2.4 µmol kg^−1^ and 110 µmol kg^−1^, respectively, at station 112. This is consistent with dissimilatory Fe and Mn reduction by microbial activity during organic matter remineralization. Organic carbon content was consistent downcore, ranging between 1.40 and 1.92%. Organic carbon δ^13^C also did not show downcore changes (figure 5), varying by only approximately 1‰ throughout the entire profile.

**Figure 6.**
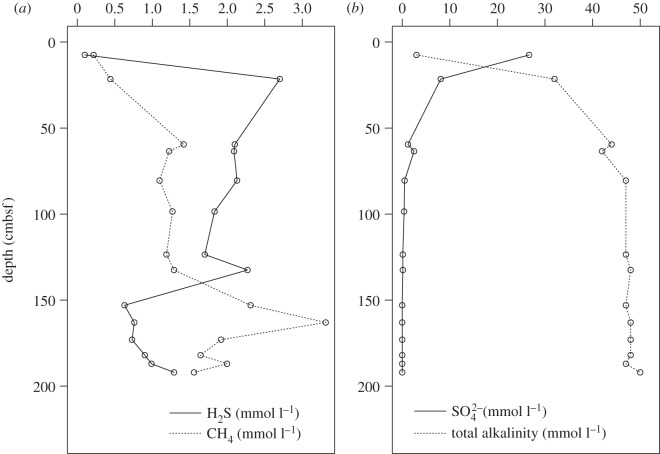
JC42 Gravity core profiles. X scale varies.

Pore water concentrations of sulfate decreased downcore from the sediment surface, with concomitant increase in sulfide and TA in both the short megacores (figure 5) and the deeper gravity core samples (figure 6). The rapid increase in dissolved methane with depth, as observed in megacores JC55 112 and 117 below 20 cmbsf (figure 5), and particularly in deeper sections of the gravity core (approx. 140–160 cmbsf), are indicative of methane production at depth (figure 6).

### 4.5. Authigenic carbonates

XRD analysis of the superficially amorphous, brittle, yellow deposit on the valves of *Axinulus antarcticus* determined that these deposits were comprised predominately of the carbonates, aragonite and dolomite. A reference sample of freeze-dried sediment did not contain either of these carbonates and the strongest signal was of quartz. One shell deposit sample also contained small signals from mica and chlorite, presumably a contamination from surrounding sediment. Although the carbonate deposits were generally located on specific areas of shell (towards the dorsal margin), they were not consistent in shape and area, indicating that they were not precipitated as part of shell growth.

## 5. Discussion

### 5.1. Community composition

Cirratulid polychaetes were by far the most numerous of any macrofaunal family, comprising 27–45% of abundance. Although a cosmopolitan family of deep-sea polychaetes (source: OBIS), their presence has also been documented at several seeps [20,36,43,69], as well as oxygen minimum zones [69] and a sedimented vent in the Bransfield Strait [45]. The presence, and high abundance, of tubificid oligochaetes and thyasirid bivalves could be indicative of reducing conditions as both of these families are known from other methane seeps [36,37,73–78].

**Figure 7.**
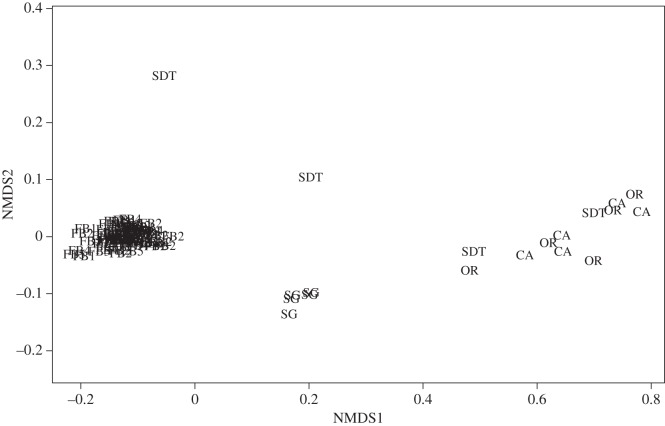
Class/Order level multidimensional scaling (MDS) plot of South Georgia macrofauna composition compared to cold seep and background sediments. CA, California margin seeps [69]; FB, FoodBancs, WAP [70]; OR, Oregon margin seeps [69]; SDT, San Diego Trough seeps [68]; SG, South Georgia (this study).

No differences were observed in assemblage composition between sites, indicating that the geochemical differences apparent in figure 5 were insufficient to influence assemblage structure. The density of *Aphelochaeta* spp., *Axinulus antarcticus* and tubificid oligochaetes accounted for 62–72% of the total and were consistently highly dominant, indicative of reducing conditions at all sites [36], supporting our suggestion of macrofaunal assemblages structured by sediment geochemistry. Faunal assemblages were compared with others from the West Antarctic Peninsula (WAP) and from seeps in the Pacific [68–70]. Assemblage composition was clearly different, at class/order level to the WAP and Pacific margin seeps (PERMANOVA, *p* = 0.001; figure 7) [70]. Polychaetes were the dominant class at the WAP and South Georgia (71.3 and 64.1%, respectively) but the South Georgia assemblages contained much higher proportions of oligochaetes and bivalves. Species-level discriminated polychaete assemblages from the WAP [70] showed very high dominance (26–70%) of a single spionid species, *Aurospio foodbancsia* [79] in contrast to the dominance of cirratulid polychaetes in South Georgia samples. Spionid polychaetes were comparatively absent from our sites (0.66% of total abundance, approx. 1% of polychaetes), represented almost entirely by *Spiophanes kroeyeri*. Samples from comparable depths (300–500 m) around the South Sandwich Islands [80] were different to the South Georgia assemblages, being instead dominated by molluscs and malacostracans, with annelids comprising a relatively small component of the assemblages. These samples, while not directly comparable owing to differences in sampling techniques (collected by epibenthic sled, as opposed to megacoring), provide evidence of differences between South Georgia and other shelf areas of the East Scotia back-arc basin at several taxonomic levels and support the suggestion that these sediments represent a continuum between seep and background sediments.

Remotely operated vehicles were deployed during JC42 (ROV *Isis*) and JC55 (Seabed High-Resolution Imaging Platform) and undertook visual surveys of areas of interest, as identified by shipboard echo sounders (EK60) and elevated methane levels in the water column, but no aggregations of microbial mat or seep-endemic fauna were observed [15,16]. Cold seep sites in the Sea of Okhotsk were also not inhabited by seep-endemic fauna shallower than 370 m depth [38] and so the absence of seep-endemic fauna from South Georgia sediments may reflect depth-related patterns in assemblage structure.

### 5.2. Trophic structure and carbon sources

Most metazoan isotope ratios were characteristic of sediment organic matter (δ^13^C_org_ −23.40‰), which is presumed to be predominately surface-derived OM, with limited *in situ* production. Estimates of the δ^13^C signatures of surface particulate organic carbon (POC) at this latitude approximately range between −29 and −24‰ [81]. Even in *Axinulus antarcticus*, where authigenic carbonates suggestive of AOM were directly observed, δ^13^C signatures of tissue were not indicative of assimilation of MDC (mean δ^13^C −20.10‰, figure 5). The lack of a strong MDC signal in metazoan tissue indicates that methanotrophy was not a significant contributor to the macrofaunal food web and we suggest that the methane flux was insufficient to support dense aggregations of methanotrophic bacteria at the sediment surface. This is similar to Californian seep sediments (depth 520 m) where macrofauna had an estimated 0–5% dietary contribution of MDC [30]. It is probable that, given the relatively high concentration of sulfate in the upper layers of the sediment and the relatively high rate of methane depletion between 20 and 30 cmbsf (figure 5), the majority of AOM activity occurred deeper than the range of the metazoans sampled here (0–10 cmbsf). This is consistent with the absence of microbial mats or aggregations of seep-endemic fauna observed during video transects of the area [13,15,16]. The sub-surface depletion of methane concentration (figure 5) emphasizes the role of microbial processes in sub-surface methane cycling, limiting potential atmospheric emissions.

Polychaetes of the order Phyllodocida (e.g. alciopids, hesionids, nephtyids and polynoids) were among the taxa with the highest δ^15^N values (more than 9‰, figure 3), indicating that these taxa occupied a higher trophic level. These taxa are all characterized by high activity (large locomotory structures) and mouthparts capable of a predatory lifestyle. However, syllids (*Sphaerosyllis hirsuta* and *Exogoninae (parexogone) tasmanica*) that also possess morphology adapted to carnivory, did not have the same δ^15^N enrichment (mean 6.12‰) and also had low δ^13^C and δ^34^S values (figure 3; [4]). This suggests that these taxa might have derived some of their organic matter from bacterial MDC, consistent with relatively low δ^13^C and δ^15^N values [21,29]. *Sphaerosyllis* and *Exogoninae* are common on carbonates at Costa Rica [43] and Hydrate Ridge seeps [82] and in Chile Margin hydrothermal sediments (Thurber *et al.* unpublished data, cited in [36]).

Interestingly, some of the highest δ^15^N values belonged to tubificid oligochaetes (mean 10.58‰ ± s.d. 0.28, figure 3) and an unidentified species of echiuran (mean 10.28‰ ± s.d. 0.01, figure 3), consistent with observations of a morphologically similar species of echiuran at a sediment-hosted hydrothermal vent in the Bransfield Strait [49]. This may indicate selective feeding strategies that favour OM that had been recycled by microbial activity, resulting in a relatively high trophic level. A visual inspection of gut content of tubificid oligochaetes (400× magnification) did not identify any remains that could be attributed to specific food sources.

Sulfur isotope ratios were the most variable of the isotopic data, suggesting diverse feeding strategies between and within taxa (figure 4). Faunal and sediment δ^34^S (Mean ± s.d. 4.25 ± 5.13 and 14.03‰ ± 0.12, respectively) were also lower than might be expected of non-methane-enriched sediments (18–20‰ [83]), indicating the influence of a relatively ^34^S-poor source. Low δ^34^S values are associated with microbial AOM [24,31] as sulfate reduction, which results in low δ^34^S signatures, co-occurs with methane oxidation. This suggests that microbial MDC was a component of the macrofaunal food web. Variability in δ^34^S signatures was unsupported by carbon isotopic measurements, which showed relatively little variation and there is a clear disparity between carbon and sulfur isotope composition. This highlights the importance of using multiple SIA to elucidate trophodynamics. The disparity between sulfur and carbon isotopic measurements could have resulted from differences in carbon and sulfur processing within the biota or as a result of the highly variable isotopic fractionation associated with sulfate–sulfide exchanges (sulfate reduction and sulfide oxidation) [31]. Additionally, it is possible that the relative contribution of AOM to total organic carbon and total organic sulfur may have been different and thus contributing to the differences in ranges observed, though this cannot be assessed from the available data.

Sediment organic carbon δ^13^C was generally lighter than most of the faunal measurements (difference in means δ^13^C of 3.90‰; figure 3) and may have been a mix of surface-derived material and MDC. The relatively heavy faunal signatures probably resulted from a preservation/treatment effect rather than an additional, unknown source of organic matter. Isotopic shifts may have arisen from ethanol preservation or the fact that sediment organic carbon samples were acidified but faunal samples were not. Untreated sediment samples had δ^13^C values that were 0.66‰ greater than acidified sediment samples [83]. Only faunal samples preserved in ethanol were selected to minimize any faunal preservation effects between different samples. However, ethanol preservation can still impact faunal signatures [84] although this effect can be very variable. A review of isotope shifts associated with preservation effects demonstrated that storing samples in more than 70% ethanol significantly increased or decreased carbon isotopic signatures in four of eight published studies. Nitrogen isotopic signatures were significantly affected in three out of five published studies. Given the variable response to ethanol preservation, it is difficult to correct isotopic signatures but it is likely that preservation effects may have artificially altered some individual faunal isotopic signatures.

### 5.3. Phospholipid fatty acids

Although it can be difficult to ascribe PLFAs to specific microbial groups or processes, some compounds are indicative of certain groups [56], especially when they occur in high relative abundance. For example, although the abundant PLFAs C16:1ω7c and C18:1ω7 may be synthesized by a number of groups, including phytoplankton and bacteria [55,58], they occurred in very high abundance in symbiont-bearing species at hydrothermal vents and seeps, and are linked to sulfide oxidation [61,85–89]. PLFAs attributed to sulfate reduction and sulfide oxidation at seeps [86] accounted for 9.3 and 27.7% of the total abundance respectively. Biomarkers specific to phytoplankton groups, like 20:5ω3 in diatoms [90], occurred in relatively low abundance (approx. 5% of the total) though this is not to say that the contribution of photosynthetically derived OM was this low, since other PLFAs are likely to have come from phytoplankton [55,90]. Photosynthetic PLFAs will also have decayed during sinking and are inevitably underestimated in such samples.

MUFAs in particular are associated with cold seeps [35,54,56,87] and three of the four most abundant FAs were MUFAs, collectively accounting for 41.24% of the total composition. While these FAs may not be exclusive to methanotrophic bacteria [55], their high relative abundance is evidence of active (or very recently active) methanotrophy in the upper layers of sediment (0–1 cm). Carbon isotopic signatures of PLFAs were generally quite similar to those of reducing sediments in the Gulf of Mexico [91]. MUFA δ^13^C ranged between −30.73 and −24.55‰ suggesting either a very heavy source of methane (compared with biogenic methane), or that these FAs were produced from a combination of methanotrophy and heterotrophic metabolism. Methane with similarly heavy isotopic values is more commonly associated with a hydrothermal origin [92], hence it is unlikely that methane was the sole source of carbon in MUFAs without a very substantial metabolic fractionation effect.

### 5.4. Geochemistry

Sulfate showed a steep decline between 0 and 50 cmbsf (figure 6), and there was a concomitant increase in H_2_S and alkalinity indicating sulfate reduction was active at these depths. The fact that the sulfate depletion was accompanied by an increase in pore water methane concentrations with depth suggests that AOM may have been active. The sulfate depletion is relatively rapid compared with methane-containing slope sediments on the Norwegian, Gulf of Mexico, Chilean and Argentine margins, where complete depletion of sulfate did not occur above 100, 250, 350 and 400 cmbsf, respectively [4,93–95]. These sites generally had comparable or higher methane concentrations below the sulfate reduction zone than the South Georgia shelf. These pore water profiles were similar to other sediments where AOM has been directly observed but the approximate depth of complete sulfate depletion was shallower on the South Georgia shelf than elsewhere [4,93,94]. Sediments with little to no methane flux showed very little decline in pore water sulfate concentrations in the top 5 m of sediment [93]. Oxygen penetration depth (figure 5) was less than 2 cmbsf, suggesting that aerobic oxidation of methane was probably very limited.

Despite a relatively steep depletion in methane concentrations between approximately 20 and 30 cmbsf, we did not observe a change in the δ^13^C of sediment organic carbon (figure 5) that might have resulted from significant rates of methanotrophy. This was probably owing to the small quantities of pore water methane relative to the total mass of organic carbon. We estimate that, if entirely converted into POC, this amount of methane would only have changed the δ^13^C_org_ by between 10^−2^ and 10^−14^‰ in each core section, depending upon the isotopic signature of the methane which was unknown, but estimated between approximately −80 and −50‰ [96]. This potential change is far less than is observable, given the variability in the data and is comparable to δ^13^C_org_ profiles at Gulf of Mexico cold seeps [97].

### 5.5. Authigenic carbonates

Authigenic carbonates have been widely documented at methane seeps, since AOM increases the concentration of bicarbonate in pore waters (figure 5; [6]) and favours *in situ* precipitation [24,49,98–100]). Carbonate production at methane seeps can result in several different forms, both low- and high-Mg content, including calcite, aragonite, dolomite and ikaite [2,43,101–103] and samples of ikaite were previously found on the southwest South Georgia margin [41]. Our observations were of small, localized carbonate crusts on the shells of *Axinulus antarcticus*, but not on other bivalve species or in the ambient sediment from the area. These observations are in contrast with previous descriptions of large carbonate precipitations within the sediment reported at other seeps [2,42,104–107].

Exterior calcification or encrusting material is known from other bivalve species. For example, some species in the family Veneridae precipitate micrometre scale, needle-shaped, aragonite formations on their periostracum [106,108]. Lucinid species, known from chemosynthetic ecosystems, can also precipitate unusual periostracal features, as arrays of scales [109]. Inspection of *A. antarcticus* and other bivalve species present (e.g. *Propeleda* sp. Nuculanidae) under a scanning electron microscope did not reveal similar structures (electronic supplementary material, S4).

Mineral deposits have also been associated with *Montacuta ferruginosa* (Montagu, Montacutidae), in this case a phosphorus-rich ferric mineral that forms part of an epibiotic biofilm [110]. These deposits were attributed to a γ-proteobacterial community, using iron, and were not linked to methanotrophy or AOM, but were similar to hydrothermal vent communities [111–113]. Microbial compositional data were not available here but given the elevated levels of methane, the AOM-associated carbonates present and the low δ^34^S signature of *Axinulus* (relative to sediment), it seems likely that these precipitates were also microbially mediated [113]. We suggest that the feeding behaviour of *A. antarcticus* enhances local fluid flow and thus favours precipitation of carbonates, potentially facilitated by a consortia of anaerobic methanotrophic bacteria [106].

### 5.6. Conclusion

Macrofaunal assemblage composition was indicative of reducing conditions but the incorporation of MDC was apparently very limited to both microbial and metazoan assemblages, similar to off-seep and near-seep areas from other studies. We highlight a disparity between carbon and sulfur isotopic measurements that emphasizes the need to use multiple isotopes in the study of trophodynamics in chemosynthetic ecosystems. Geochemical signatures were consistent with weak methane seepage and we demonstrate the role of sub-seafloor methane consumption, preventing methane emissions into the bottom water.

## Supplementary Material

S1 - Macrofaunal assemblage composition (quantitative cores only, abundance per m2) and stable isotopic data. A = 0 – 5 cmbsf, B = 5 – 10 cmbsf

## Supplementary Material

S2 - PLFA composition and abundance

## Supplementary Material

S3 - Geochemical profiles (megacores and gravity core)

## Supplementary Material

S4 - SEM Micrographs of the valves of several bivalve species. A = Axinulus antarcticus; B = Propeleda longicaudata and C = Ennucula sp. (cf. georgiana).

## Data accessibility

The data presented in this paper are accessible as electronic supplementary material that accompanies this article.
