# Supplementary figures and images for: Geochemistry, faunal composition and trophic structure in reducing sediments on the southwest South Georgia margin
Source: R Soc Open Sci. 2016 Sep 28;3(9):160284. doi: 10.1098/rsos.160284 (PMC5043311; doi:10.1098/rsos.160284)

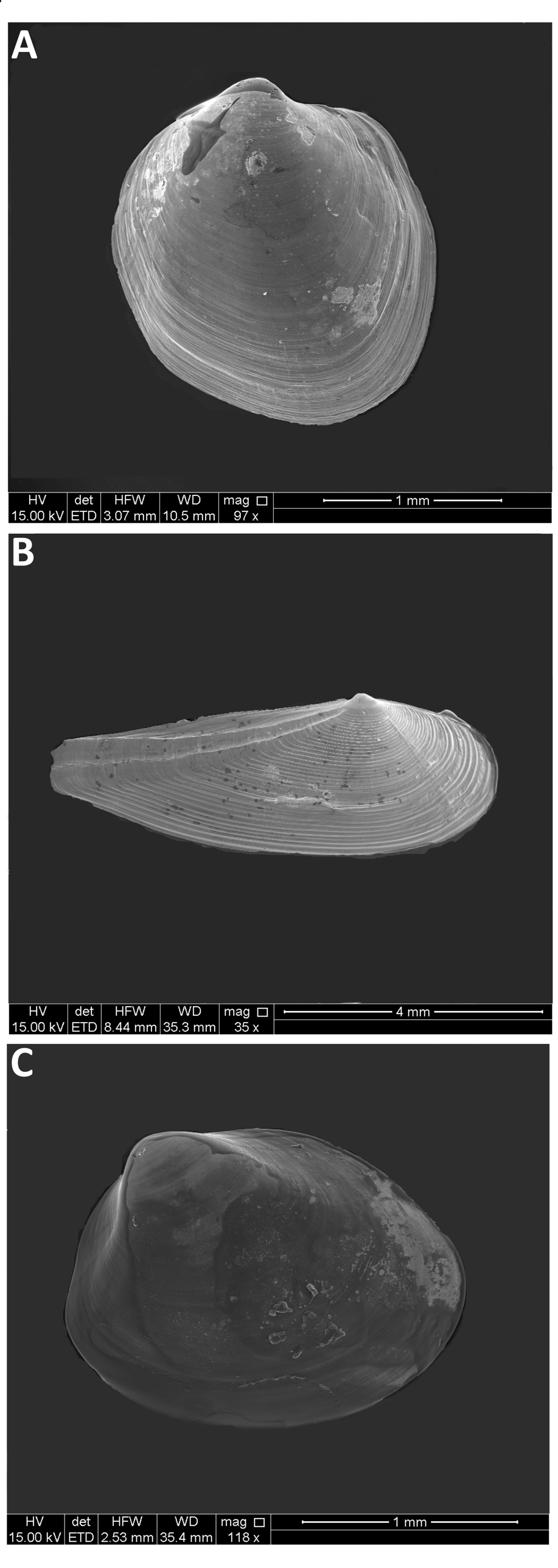

Supplement: S4 - SEM Micrographs of the valves of several bivalve species. A = Axinulus antarcticus; B = Propeleda longicaudata and C = Ennucula sp. (cf. georgiana). [file rsos160284supp4.tif]
